# T5. LURASIDONE AND RISK FOR METABOLIC SYNDROME IN PATIENTS WITH SCHIZOPHRENIA: A COMPREHENSIVE DATABASE ANALYSIS

**DOI:** 10.1093/schbul/sby016.281

**Published:** 2018-04-01

**Authors:** Michael Tocco, Andrei Pikalov, Jay Hsu, Josephine Cucchiaro, John W Newcomer, Antony Loebel

**Affiliations:** 1 Sunovion Pharmaceuticals, Inc; 2 Florida Atlantic University Charles E. Schmidt College of Medicine

## Abstract

**Background:**

Patients with schizophrenia are at increased risk for developing metabolic syndrome, with an estimated prevalence of approximately 35–50% (Correll et al. Psychiatr Serv 2010;61:892–98; Vancampfort et al. World Psychiatry 2015;14:339–47). Treatment with atypical antipsychotic medications have been shown to increase rates of metabolic syndrome, with differences observed among antipsychotic agents, most notably in propensity for weight gain: higher for olanzapine, clozapine, and iloperidone; intermediate for quetiapine, risperidone, and paliperidone; and lower for amisulpride, aripiprazole, asenapine, lurasidone, and ziprasidone (Leucht et al. Lancet 2013;382:951–62). Independent of weight gain, atypical antipsychotics also appear to have direct effects on lipid metabolism and glucose regulation. The aim of this safety analysis was to assess the effects of treatment with lurasidone on metabolic syndrome risk in patients with schizophrenia.

**Methods:**

Changes in the rate of metabolic syndrome during treatment with lurasidone (40–160 mg/d) versus active comparators (olanzapine, quetiapine, risperidone) were analyzed using pooled short-term data from 3 randomized, double-blind, placebo-controlled studies; long-term data from 2 active-controlled studies; and switch data from 2 open-label extension studies. Metabolic syndrome was defined based on the National Cholesterol Education Program criteria (NCEP ATP III; 2005 revision).

**Results:**

In short-term studies, risk of treatment-emergent metabolic syndrome was similar for patients in the lurasidone and placebo groups (odds ratio [OR]=0.97; week 6 LOCF-endpoint); and was significantly greater for patients in the olanzapine (OR=2.68; P<0.001) and quetiapine (OR=3.70; P<0.001) groups compared to placebo. In long-term studies, risk of treatment-emergent metabolic syndrome after 12 months was significantly lower for lurasidone compared with risperidone (OR=0.374; 95% CI, 0.180–0.774; P<0.01) and non-significantly lower for lurasidone compared with quetiapine XR (OR=0.267; 95% CI, 0.040–1.806; P>0.05). In open-label switch studies, the rate of metabolic syndrome decreased in patients switched to lurasidone after 6 weeks of treatment with olanzapine or 12 months of treatment with risperidone.

**Discussion:**

In this comprehensive analysis of the lurasidone clinical trial data base, treatment with lurasidone (40–160 mg/d) was not associated with the development of metabolic syndrome in patients with schizophrenia. Rates of metabolic syndrome increased in patients treated with olanzapine, risperidone, and quetiapine XR.

